# Trends in the disease burden of thyroid cancer among adolescents and young adults: A comparative study of China and global estimates (1990–2021)

**DOI:** 10.1371/journal.pone.0333373

**Published:** 2025-10-14

**Authors:** Jiahui Qian, Shuo Qi, Rui Hu, Miao Zhang, Zhi Chen, Zhiguo Ding

**Affiliations:** 1 Dongzhimen Hospital, Beijing University of Chinese Medicine, Beijing, China; 2 Beijing University of Chinese Medicine, Beijing, China; 3 Thyropathy Hospital, SUN Si Miao Hospital, Beijing University of Chinese Medicine, Tongchuan, Shaanxi, China; 4 Hainan Medical University, Haikou, Hainan, China; National Center for Chronic and Noncommunicable Disease Control and Prevention, Chinese Center for Disease Control and Prevention, CHINA

## Abstract

**Objective:**

This study aimed to assess temporal trends, epidemiological features, and sex differences in the thyroid cancer burden among adolescents and young adults, globally and in China, from 1990 to 2021, using the Global Burden of Disease 2021 data. We projected China’s future burden through 2041, to support precision prevention strategies.

**Methods:**

Data on the incidence, prevalence, mortality, and disability-adjusted life years of thyroid cancer in AYAs were extracted from the Global Burden of Disease 2021 database. Age-standardized rates and estimated annual percentage changes were calculated. Joinpoint regression was used to detect shifts in trends. An age-period-cohort model was used to quantify the effects of age, period, and birth cohort on the incidence. Decomposition analysis was used to evaluate the contributions of population growth, aging, and epidemiological changes. A Bayesian age-period-cohor model projected future trends for China. Furthermore, we conducted a stratified analysis by sex to investigate the heterogeneity in the evolution of disease burden between the Chinese and global populations.

**Results:**

Between 1990 and 2021, China experienced a rapid increase in the incidence and prevalence of thyroid cancer among adolescents and young adults; the age-standardized incidence rate increased by 152.6%, far exceeding the global average. Mortality and disability-adjusted life years declined, forming a pattern of high incidence, low mortality, and moderate disability. This increase was primarily driven by epidemiological transitions. In terms of sex differences, Males exhibited a sharper increase in both incidence and disability-adjusted life years than females, indicating growing sex-based disparities. Notably, the burden in China began increasing earlier and more rapidly than global trends, particularly in males, whose incidence continued to increase even as global rates stabilized. Age-period-cohort model analysis further revealed that, among the adolescent and young adult population in China, the incidence of thyroid cancer increased significantly with advancing age, the incidence risk potentially increased after 2010, and more recent birth cohorts born after 1980 also exhibited an upward risk trend. However, despite these patterns, neither the period nor cohort effect reached statistical significance. Finally, Bayesian age-period-cohort projections suggested that the incidence and prevalence will continue to increase over the next 20 years, while mortality will remain stable, and disability-adjusted life years will decline slightly.

**Conclusions:**

Over the past three decades, the burden of thyroid cancer among adolescents and young adults in China has increased at a substantially accelerated rate compared to global trends, with a pronounced widening of the sex gap in disease burden. While Chinese female patients have continued to show improvements in prognosis and relative burden measures, male patients have experienced a marked rise in both incidence and disability-adjusted life years, accompanied by a consistent decline in prognostic outcomes. These findings underscore the need for sex-specific strategies: for males, prioritizing the identification and mitigation of modifiable risk factors to curb the rising burden and improve prognosis; for females, consolidating effective diagnostic and therapeutic practices while minimizing overdiagnosis. Gender-sensitive approaches, aligned with precision prevention, may help address the growing burden.

## Introduction

Adolescents and young adults (AYAs; ages 15–39 years) represent a distinct subgroup whose cancer burden is frequently overlooked in global estimates, despite possessing unique epidemiological patterns, clinical care needs, and socioeconomic impacts [1]. Thyroid cancer (TC) accounts for over 90% of endocrine malignancies [2] and is the most common head and neck tumor in adults [3]. Globally, TC is the 13th most common cancer and the 6th most common among females, and its burden is evolving markedly, with a disproportionately large share observed in AYAs [4,5]. In the United States, TC is the most frequent solid malignancy in AYAs aged 16–33 years, with an incidence exceeding that of the next most common cancer (gonadal germ cell tumors) by more than 40%, and has become the leading malignancy in females in this age group [6]. The AYA TC incidence has increased steadily since 1990 [7], and in certain regions, this elevated incidence has driven much of the overall increase in AYA cancer rates [6], underscoring the urgent need for targeted disease prevention and control in this population.

Young-adult patients with TC exhibit pronounced clinical heterogeneity that warrants close attention. Many cancers diagnosed in AYA patients differ significantly from those diagnosed in older age groups in terms of prognosis and survival rates, tumor biology, and risk factors [8]. Most patients with TC have a favorable prognosis [9], and the disease-specific survival for differentiated thyroid carcinoma in young adults approaching 98% by age 40 [6]. Although AYA patients generally have a better prognosis than older individuals [8], their tumor behavior is distinct. Compared with adults, AYA patients more often present with larger tumors (only 18% are microcarcinomas of <1 cm), greater invasive potential, and a higher incidence of extensive cervical lymph node and distant metastases [10,11]. Postoperative radioiodine therapy (RAI), while reducing the recurrence risk, poses unique challenges for AYAs. Individuals under 25 years of age experience elevated standardized incidence ratios for salivary gland cancer (34.12) and leukemia (3.98) [12], and RAI can induce temporary gonadal dysfunction [13]. Furthermore, long-term survivors shoulder significant economic and psychological burdens from lifelong thyroid hormone replacement therapy and ongoing surveillance; patients aged 20–34 years have a 3.5‐fold higher risk of bankruptcy than their peers [14].

Despite being a paradigmatic example in oncology research, AYA TC is often subsumed under the broad “adult” or “pediatric” categories, resulting in a paucity of age‐specific data on molecular characteristics, therapeutic responses, and social needs, especially in China. This blind spot may hinder the development of precise diagnostics and interventions. Approximately 76% of AYA TC cases are detected incidentally on imaging, suggesting that overdiagnosis may inflate incidence estimates, whereas socioeconomically disadvantaged AYAs face delayed diagnoses and advanced-stage presentations [6,15]. This paradox underscores the need for large-scale epidemiological studies to determine the true disease burden and its drivers.

According to 2022 global cancer statistics, China accounted for 56.8% of global TC cases in 2022 (466,118/821,173), making it the nation’s 3rd most common malignancy. Among the top-10 malignancies by incidence in China, TC has shown the fastest growth since 2016, with cases surging 149.8% in males and 123.6% in females [16,17]. China’s prominent position in the global TC burden forms a sharp contrast with the severe scarcity of key epidemiological data for its vast domestic AYA population. Therefore, comparative analysis of Chinese AYA TC within a global context is essential to understand its unique patterns, bridge knowledge gaps, and guide precision prevention and control.

By analyzing data from the Global Burden of Disease (GBD) study, this study aimed to characterize the epidemiological features of TC among AYAs, both globally and in China. Specifically, we sought to identify the distinct epidemiological patterns of China within the global landscape and determine the disease burden trajectory over the next two decades. This China-specific analysis within a global context aims to establish an evidence base for precision prevention and control strategies targeting TC in Chinese AYAs.

## Materials and methods

### Data source and overall framework

The data for this study were obtained from the Global Burden of Disease Study 2021 (GBD 2021; https://vizhub.healthdata.org/gbd-results/). GBD 2021 represents a comprehensive synthesis of data from surveys, censuses, vital registration systems, and other health-related sources to estimate the incidence, prevalence, mortality, and disability-adjusted life years (DALYs). For TC among AYAs, we extracted annual estimates of the incidence, prevalence, mortality, and DALYs (with their corresponding 95% uncertainty intervals [UIs]) directly from the GBD 2021 repository.

In this study, TC was defined based on the GBD 2021 framework, which adopts the International Classification of Diseases 10th Revision (ICD-10) code C73. This classification includes all malignant neoplasms of the thyroid gland regardless of histological subtype. Therefore, the burden estimates in our analysis encompass all major histological types of TC, including papillary, follicular, medullary, and anaplastic carcinomas.

### Age-standardized rates and estimated annual percentage change

To eliminate the confounding effect of different age structures between populations, we calculated age-standardized rates (ASRs) for the incidence (ASIR), prevalence (ASPR), mortality (ASMR), and DALYs (ASDR) per 100,000 people using the GBD world standard population [18]. Temporal trends in the ASRs were quantified using the estimated annual percentage change (EAPC), which is a widely adopted metric for ASR trend analysis [19,20]. An EAPC >1 indicated a statistically significant upward trend, <−1 indicated a downward trend, and values between –1 and 1 denoted relative stability.

### Trend analysis

We applied the Joinpoint Regression Program (version 5.1.0.0) to identify inflection points—“joinpoints”—at which significant changes in the temporal trend occurred and to compute the average annual percentage change (AAPC) across the entire study period [21]. Joinpoint regression fit the segmented linear models to the log-transformed ASRs, automatically selecting the optimal number of joinpoints using permutation tests. An AAPC with a 95% confidence interval (CI), excluding zero, reflected a significant upward (CI > 0) or downward (CI < 0) trend [22].

### Age-period-cohort (APC) analysis

Given the multifactorial etiology of TC, we employed an APC model to disentangle the independent effects of chronological age, calendar period, and birth cohort on incidence trends. Analyses were performed using the web-based APC tool developed by the U.S. National Cancer Institute (NCI) using R (R Foundation for Statistical Computing, Vienna, Austria). In this framework, “age effects” captured biological aging processes, “period effects” reflected environmental and diagnostic shifts affecting all age groups simultaneously, and “cohort effects” represented generational differences in risk factor exposure. Statistical significance was assessed using two-sided Wald chi-square tests, and p-values <0.05 denoted statistical significance.

### Decomposition analysis

We used a Das Gupta decomposition analysis to quantify the contributions of demographic and epidemiological drivers to the changes in TC incidence between 1990 and 2021 [23,24]. This algebraic approach isolated the impacts of population growth, population aging, and changes in age-specific incidence rates by maintaining other factors constant, thereby estimating the individual contribution of each component to the overall change.

### Bayesian age-period-cohort (BAPC) projection

We extended our temporal analysis by implementing a BAPC model to forecast the TC incidence among AYAs in China from 2021 to 2041. In the BAPC model, age, period, and cohort effects were assigned second-order random walk (RW2) priors to ensure smooth changes over time and age. The precision parameters followed a log-Gamma prior, which controls the degree of smoothness. To assess the predictive validity of the model, we used TC data among Chinese AYAs from 1990 to 2015 to forecast the subsequent six years (2016–2021) and compare these predictions with the observed data. Midyear population projections were sourced from the World Health Organization (WHO) via the United Nations World Population Prospects database (https://population.un.org/wpp).

All modeling and visualizations were conducted in R (version 4.3.2) using the BAPC package (version 0.0.35) with ggplot2 (version 3.4.4) and dplyr (version 1.1.3) for ancillary analyses and figure generation.

### Ethics statement

This study is a secondary analysis of publicly available, de-identified data from the Global Burden of Disease (GBD) 2021 database. As such, it did not require approval from an institutional review board or ethics committee. Informed consent was not applicable, as no human participants were directly involved, and no identifiable personal data were accessed.

## Results

### Evolution of AYA TC burden in China versus global trends (1990–2021) “Dual-acceleration” in incidence and prevalence with “dual-improvement” in health outcomes

The ASR are summarized in Table 1. Between 1990 and 2021, the ASIR of AYA TC in China increased markedly from 0.57 per 100,000 (95% CI: 0.43–0.73) to 1.44 per 100,000 (1.13–1.90), representing a cumulative increase of 152.6% (EAPC = 3.23%). Over the same period, the global ranking for AYA TC incidence in China increased from 133rd to 72nd among 204 countries and territories, an upward shift of 61 places to a ranking of 18th worldwide in ASIR growth. Concurrently, the ASPR increased from 5.11 per 100,000 (3.83–6.54) to 13.11 per 100,000 (10.25–17.37), a 156.6% increase (EAPC = 3.31%). As a result, the prevalence rank in China changed from 133rd to 73rd (a 60-place change), and the global growth rate was 19th. Both ASIR and ASPR growth rates were within the top deciles globally, indicating that TC had become a priority malignant neoplasm for AYA prevention and control in China.

**Table 1:** Comparison of age-standardized incidence rates (ASR) and estimated annual percentage change (EAPC) between Chinese and global populations, 1990–2021. The table summarizes trends in thyroid cancer burden among AYAs in China and globally from 1990 to 2021. Incidence and prevalence increased substantially, especially in China, while mortality remained low. DALYs declined slightly in China but rose globally, reflecting the high-incidence–low-mortality pattern of thyroid cancer.

Measure	Location	Num_1990	ASR_1990	Num_2021	ASR_2021	EAPC_CI
**DALYs**	China	20307.44 (15721.65–25927.89)	3.71 (2.87–4.74)	17170.66 (13417.12–22425.01)	3.39 (2.65–4.42)	−0.34 (−0.46– −0.22)
**Deaths**	China	314.2 (242.83–397.66)	0.06 (0.04–0.07)	229.87 (182.65–294.47)	0.04 (0.04–0.06)	−0.87 (−0.97– −0.76)
**Incidence**	China	3130.5 (2354.02–4000.24)	0.57 (0.43–0.73)	7528.38 (5884.91–9959.27)	1.44 (1.13–1.9)	3.23 (3.07–3.39)
**Prevalence**	China	27910.96 (20942.6–35684.14)	5.11 (3.83–6.54)	68613.33 (53624.03–90863.43)	13.11 (10.25–17.37)	3.31 (3.15–3.47)
**DALYs**	Global	114722.31 (99193.33–136900.66)	5.23 (4.53–6.25)	183484.77 (149893.24–232300.94)	6.17 (5.04–7.81)	0.52 (0.48–0.56)
**Deaths**	Global	1738.15 (1506.82–2071.38)	0.08 (0.07–0.09)	2652.26 (2178.65–3314.49)	0.09 (0.07–0.11)	0.34 (0.3–0.39)
**Incidence**	Global	19268.18 (17244.73–22058.29)	0.88 (0.79–1.01)	48203.25 (40775.81–58353.46)	1.62 (1.37–1.96)	2.08 (1.98–2.17)
**Prevalence**	Global	172263.26 (154319.3–196952.88)	7.86 (7.04–8.99)	436143.74 (368967.18–527965.77)	14.66 (12.4–17.75)	2.12 (2.02–2.22)

Despite this marked increase in the burden, health outcome indicators improved. The ASMR in China decreased by 33.3%, from 0.06 per 100,000 (0.04–0.07) to 0.04 per 100,000 (0.04–0.06) (EAPC = –0.87%), and the global ASMR ranking changed from 133rd to 98th (a 35-place improvement). The ASDR decreased from 3.71 per 100,000 (2.87–4.74) to 3.39 per 100,000 (2.65–4.42) (EAPC = –0.34%), and the global ranking changed from 108th to 97th. The increasing incidence alongside decreasing mortality and DALYs highlighted the effective diagnostic and therapeutic strategies for AYAs with TC in China.

### Global “three-up, one-plateau” pattern

Worldwide, the AYA TC ASIR increased from 0.88 per 100,000 (0.79–1.01) to 1.62 per 100,000 (1.37–1.96) (EAPC = 2.08%), and the global median incidence rank shifted from 85th to 63rd. The ASPR increased by 86.5%, from 7.86 per 100,000 to 14.66 per 100,000 (EAPC = 2.12%), and the median rank increased by 49 places. The ASDR increased modestly, from 5.23 per 100,000 to 6.17 per 100,000 (EAPC = 0.52%), resulting in a 12-place decline in the median ranking. In contrast, the increase in ASMR slowed by 12.5% (EAPC = 0.34%), and the median mortality rank fell by 21 positions.

### Transnational heterogeneity in AYA TC burden

The ASIR of TC among AYAs in China increased at an EAPC of 3.23%, which is 1.55 times the global average (EAPC = 2.08%). In contrast, China showed more favorable outcome metrics than global medians: in 2021, the ASMR was 0.04 per 100,000, slightly lower than the global median of 0.05 per 100,000, and the ASDR declined over time (EAPC = –0.34%), whereas the global ASDR showed a rising trend (EAPC = 0.52%). Specifically, the global ASDR in 2021 was 6.17 per 100,000 population, while China reported a markedly lower rate of 3.39 (95% CI: 2.65–4.42).

The DALYs-to-incidence ratio in China dropped significantly from 6.51 in 1990 to 2.35 in 2021 (−63.9%), while the global ratio remained relatively stable. Similarly, China’s incidence-to-mortality ratio rose substantially from 88.8 in 1990 to 298.5 in 2021 (+236%), far exceeding the global increase from 99.1 to 164.4 (+66%). The sharp rise in ASIR and notable improvement in survival among Chinese AYAs may, in part, reflect the impact of overdiagnosis—particularly the detection of indolent or subclinical tumors that would not have caused symptoms or death.

Together, these trends outline a distinctive “high detection-low mortality-low disability” pattern of TC burden and control in China.

### Sex-specific burden of TC among AYAs in China and globally “Diverging acceleration–contrasting prognosis” in Chinese AYAs

**Fig 1:**
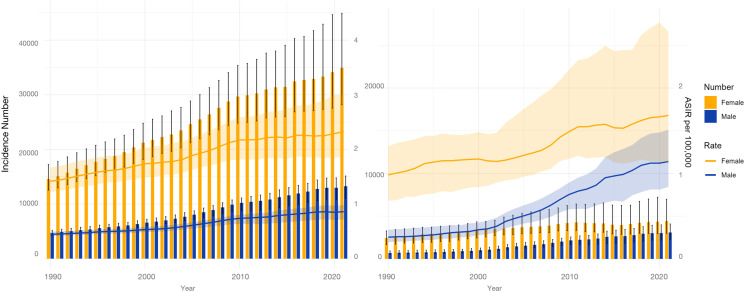
Temporal trends in thyroid cancer burden among AYAs (1990–2021): Age-standardized rates and absolute case counts. Lines represent the ASIR(per 100,000). Scale bars indicate the absolute number of cases. Males (blue), females (red); global data (left), China (right). ASIR = age-standardized incidence rate.

As shown in Table 2, Chinese AYAs exhibited a pronounced sex-dimorphic pattern characterized by “diverging acceleration–contrasting prognosis.” Between 1990 and 2021(temporal trends in Fig 1), the EAPC of incidence among male AYAs was 6.02%, which was three times that in females (EAPC = 2.01%). Cumulatively, the male ASIR increased by 392%, which was 4.3-fold higher than the 91.7% increase observed in females. This shift altered the prevalent cohort structure; male AYAs accounted for 27.6% of cases in 1990 (6,039/21,872) but 40.9% in 2021 (28,045/68,613), elevating the male-to-female ratio to 0.69:1, far above the global average of 0.38:1.

**Table 2:** Comparison of ASR and EAPC by sex between Chinese and global populations, 1990–2021.

Six	Measure	Location	Num_1990	ASR_1990	Num_2021	ASR_2021	EAPC_CI
**Female**	DALYs	China	13128.94 (9036.24–17880.3)	4.96 (3.42–6.76)	7629.02 (4988.87–12166.22)	3.06 (2–4.91)	−1.98 (−2.15– −1.8)
**Female**	DALYs	Global	77847.32 (62594.08–99125.46)	7.19 (5.78–9.15)	119298.79 (90811.22–165171.89)	8.14 (6.2–11.27)	0.32 (0.26–0.39)
**Male**	DALYs	China	7178.51 (5496.54–9571.5)	2.54 (1.94–3.38)	9541.64 (6871.82–12510.3)	3.67 (2.63–4.83)	1.67 (1.51–1.83)
**Male**	DALYs	Global	36874.99 (32605.8–42812.19)	3.33 (2.94–3.86)	64185.98 (51131.45–75764.67)	4.25 (3.39–5.02)	0.92 (0.88–0.97)
**Female**	Deaths	China	202.27 (139.14–272.44)	0.08 (0.05–0.1)	94.09 (63.59–147.93)	0.04 (0.03–0.06)	−2.73 (−2.91– −2.55)
**Female**	Deaths	Global	1166.46 (941.69–1489.51)	0.11 (0.09–0.14)	1685.9 (1289.83–2313.6)	0.12 (0.09–0.16)	0.11 (0.05–0.18)
**Male**	Deaths	China	111.93 (86.08–148.6)	0.04 (0.03–0.05)	135.78 (99.13–178.67)	0.05 (0.04–0.07)	1.26 (1.12–1.4)
**Male**	Deaths	Global	571.69 (508.73–662.63)	0.05 (0.05–0.06)	966.36 (772.94–1138.04)	0.06 (0.05–0.08)	0.79 (0.75–0.83)
**Female**	Incidence	China	2437.58 (1687.86–3275.77)	0.92 (0.64–1.24)	4435.21 (3002.99–6978.42)	1.74 (1.18–2.75)	2.01 (1.87–2.16)
**Female**	Incidence	Global	14580.38 (12560.57–17218.76)	1.35 (1.16–1.59)	34939.59 (28167.51–44916.72)	2.38 (1.92–3.07)	1.9 (1.79–2)
**Male**	Incidence	China	692.92 (532.19–934.54)	0.25 (0.19–0.33)	3093.17 (2295.7–4090.33)	1.16 (0.86–1.54)	6.02 (5.72–6.32)
**Male**	Incidence	Global	4687.79 (4290.08–5166.97)	0.42 (0.39–0.47)	13263.66 (11060.73–15128.88)	0.88 (0.73–1)	2.61 (2.52–2.71)
**Female**	Prevalence	China	21872.01 (15131.3–29375.11)	8.29 (5.73–11.14)	40567.9 (27468.62–63843.08)	15.89 (10.75–25.13)	2.08 (1.94–2.22)
**Female**	Prevalence	Global	130958.13 (112955.41–154466.62)	12.09 (10.43–14.26)	317097.88 (255787.02–407396.93)	21.64 (17.46–27.81)	1.94 (1.83–2.05)
**Male**	Prevalence	China	6038.96 (4639–8156.23)	2.14 (1.64–2.89)	28045.43 (20835.39–37108.6)	10.53 (7.79–13.96)	6.17 (5.87–6.47)
**Male**	Prevalence	Global	41305.13 (37830.98–45473.37)	3.73 (3.41–4.1)	119045.86 (99292.01–135772.25)	7.89 (6.58–8.99)	2.69 (2.59–2.78)

The disparities in health outcome metrics had widened. The male ASDR increased by 44.5% (EAPC = 1.67%), whereas the female ASDR decreased by 38.3% (EAPC = –1.98%). Mortality trends diverged strikingly: the ASMR increased by 25.0% (from 0.04 to 0.05 per 100,000) in males, in stark contrast to a 50.0% reduction among females (from 0.08 to 0.04 per 100,000). This sex-specific prognostic reversal was unique to the global stage.

### Global sex-specific burden distribution

Globally, the absolute burden (Fig 1) remained higher in female AYAs; the 2021 ASIR was 2.38 versus 0.88 per 100,000 and the ASPR was 21.64 versus 7.89 per 100,000, an approximately 2.7-fold advantage. However, temporal dynamics favored male AYAs. The male incidence EAPC of 2.61% outpaced the female EAPC of 1.90%, and male ASDR growth (EAPC = 0.92%) exceeded that of females (EAPC = 0.32%). The male mortality acceleration (EAPC = 0.79%) was 7.2 times that of females (EAPC = 0.11%), indicating a worsening prognosis for male AYAs and posing an emerging global public health challenge.

### Transnational heterogeneity in AYA TC burden

#### Mortality trend divergence.

China outperformed the global norms in outcome improvements. The Chinese AYA female ASMR decreased by 50.0% from 1990 to 2021, whereas the global female ASMR increased by 9.1%. Similarly, the Chinese female ASDR declined by 38.3% (EAPC = −1.98%), compared to a 13.2% global increase (EAPC = 0.32%).

#### Excessive increase in Chinese male burden.

**Fig 2:**
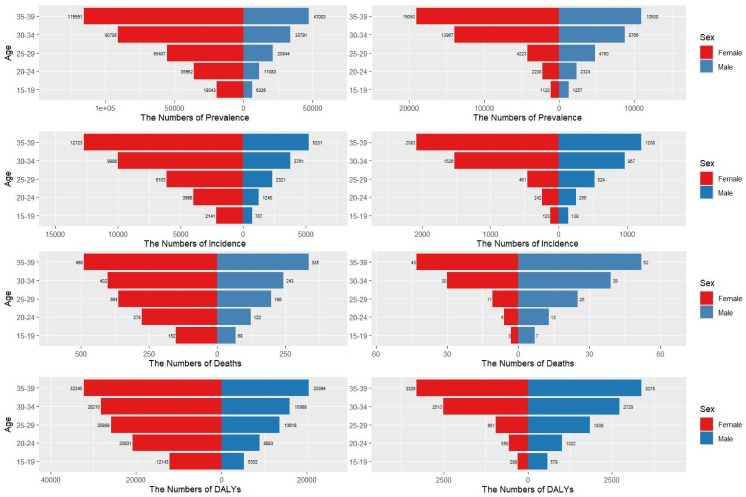
Sex-stratified incidence, prevalence, mortality, and DALYs in China (right) and globally (left). Data represent the absolute incidence, prevalence, mortality, and DALYs. DALYs = disability-adjusted life years.

As shown in Fig 2, temporal trends reveal that Chinese male AYAs exhibited a “catch-up effect.” Their ASIR growth (EAPC = 6.02%) was 2.3 times the global average for males (EAPC = 2.61%) and three times the rate of Chinese females. The male-to-female incidence ratio increased from 0.28:1 in 1990 to 0.69:1 in 2021, which was 81.6% higher than the global ratio of 0.38:1, signifying a fundamental shift in the burden by sex. In terms of disability, the Chinese male ASDR growth (EAPC = 1.67%) surpassed the global male EAPC of 0.92%. By 2021, Chinese male DALYs comprised 19.7% of the global male AYA TC burden, which was up from 4.2% in 1990, representing a 4.7-fold increase. This male-driven surge contrasted sharply with the global female-dominated distribution (ASIR sex ratio of approximately 2.7:1).

### Decomposition analysis of AYA TC burden: China versus global

**Fig 3:**
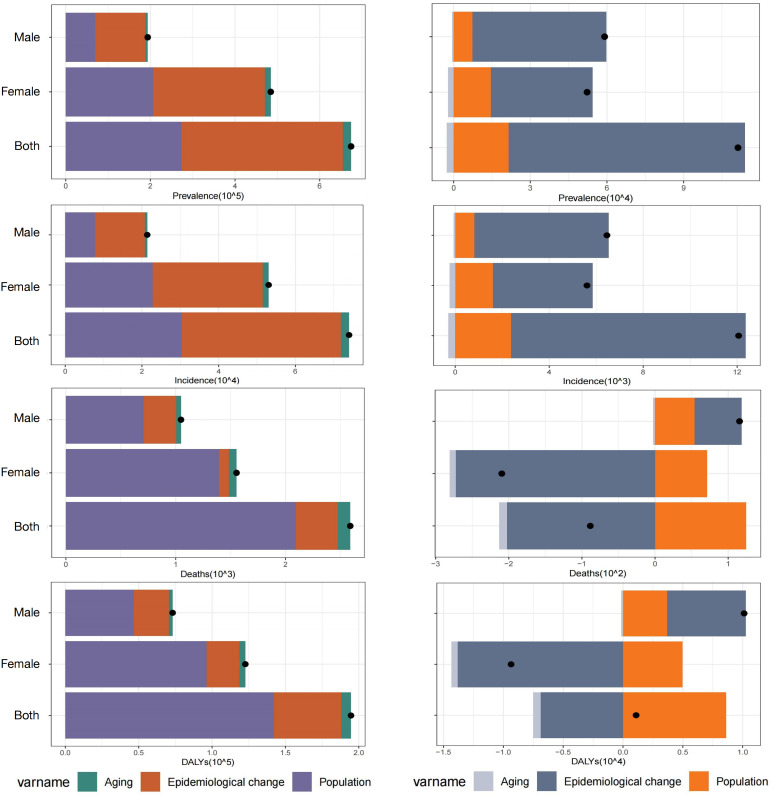
Sex-stratified decomposition of thyroid cancer burden in 2021: incidence, prevalence, DALYs, and mortality. (left: global data; right: China).

This study systematically compared the contributions of demographic and epidemiological drivers to changes in the TC burden among AYAs in China and worldwide, stratified by sex(as shown in Fig 3). Overall, the shifts in China’s burden were driven overwhelmingly by epidemiological changes, whereas global trends reflected a more balanced interplay between population growth and epidemiological risk, with marked sex differences.

#### Incidence and prevalence.

Worldwide, increases in the AYA TC incidence and prevalence were driven primarily by changes in age-specific rates (56.0% incidence growth and 56.5% prevalence growth) and population expansion (41.1% and 40.6%, respectively), with a minimal contribution from population aging (approximately 2.9% for both).

In contrast, the increases in the incidence and prevalence in China were dominated by epidemiological shifts (82.7%, incidence; 83.0%, prevalence), likely reflecting enhanced detection capabilities, such as widespread ultrasound screening. Population growth (19.8% and 19.4%, respectively) played a lesser role, and aging exerted a small negative effect (approximately 2.4%). For sex stratification, among Chinese AYAs, epidemiological changes accounted for >88% of the male incidence and prevalence growth (versus approximately 61% globally) and for approximately 76% of female growth (global female figures showed more equal contributions from demographic and epidemiological drivers).

#### Mortality and DALYs: off setting population expansion pressures.

Globally, the increasing AYA TC mortality was driven predominantly by population growth (80.8%), with modest impacts from epidemiological changes (14.8%) and aging (4.4%), suggesting a limited offset from diagnostic or treatment improvements. In contrast, epidemiological improvements in China provided a large contribution (−228.4%), which reflected substantial declines in age‐specific mortality, while population growth (140.7%) increased the total deaths, and aging (−12.3%) provided further mitigation.

#### Sex differences.

In China, the 129.9% decline in female AYA mortality was attributable to epidemiological gains (97%) and aging (3%); male AYA mortality increased by 55.6%, driven entirely by adverse epidemiological factors (e.g., later stage at diagnosis). Globally, 94.3% of female mortality increases were due to demographic shifts, whereas only 28.2% of male mortality increases were associated with epidemiological risks.

Worldwide increases in DALYs among AYAs were largely fueled by population expansion (73.1%). In China, epidemiological improvements contributed a substantial negative share (−627.3%), yet population growth (786.6%) remained the principal upward force.

#### Sex stratification.

Chinese male DALYs increased under the combined effects of population growth (36%) and epidemiological deterioration (65%), whereas female DALYs were controlled by epidemiological gains (−147%) despite demographic increases (53%). Globally, 79% of female DALY growth was driven by population expansion, and increases in male DALY reflected both demographic (63.9%) and epidemiological (32.7%) contributions.

### Divergent evolution of AYA TC burden in China and globally: Joinpoint analysis

**Fig 4:**
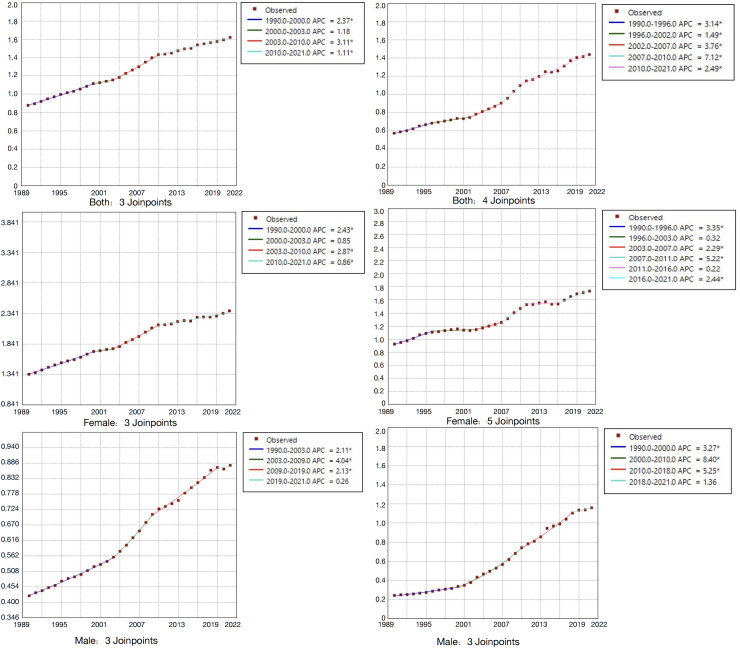
Joinpoint analysis of thyroid cancer incidence in global and Chinese AYA populations (left: global data; right: China).

As shown in Fig 4, from 1990 to 2021, the incidence among AYAs showed a phased upward trend, both globally and in China. The increase occurred earlier in China than globally, with a greater magnitude and more pronounced fluctuations. The AAPC in China significantly exceeded the global rate, indicating a clearly worsening disease burden. Sex-specific analysis revealed that the incidence in Chinese males continued to rise rapidly, diverging from the global trend of stabilization, while the incidence among Chinese females followed a cyclical “stagnation-rebound” pattern. These findings highlight the need for sex-sensitive, sustainable, long-term preventive strategies.

#### Global and Chinese incidence rates showed phased increases, with the growth in China starting earlier, fluctuating more, and contributing to a greater cumulative burden.

Joinpoint regression analysis showed that from 1990 to 2021, the global incidence of joinpoints experienced several distinct growth phases: an initial rapid increase from 1990 to 2000 (APC = 2.37%), a slowdown from 2000 to 2003 (APC = 1.18%), reacceleration from 2003 to 2010 (APC = 3.11%), and a subsequent deceleration after 2010 (APC = 1.11%).

In contrast, China experienced an earlier onset and more intense trajectory: rapid growth from 1990 to 1996 (APC = 3.14%), a modest slowdown from 1996 to 2002 (APC = 1.49%), renewed acceleration from 2002 to 2007 (APC = 3.76%), and a very rapid increase from 2007 to 2010 (APC = 7.12%). The rate of increase slowed from 2010 to 2021 (APC = 2.49%) but remained substantially higher than the global level during the same period (APC = 1.11%).

The AAPC of Chinese AYAs was 3.1%, which was 55% higher than the global average of 2.0%, suggesting a more severe and accelerating epidemic in China. Notably, while global growth rates slowed since 2010, China sustained a relatively high increase (APC = 2.49%), indicating ongoing pressure on disease control efforts. The combination of earlier high-growth periods (e.g., APC = 7.12% during 2007–2010) and subsequent persistent increases led to a rapid expansion of the cumulative disease burden.

#### Incidence among global males tended to stabilize, whereas Chinese males experienced sustained and rapid increases, differing from the global trend.

Sex-stratified analysis indicated a notably sharp upward trend among Chinese male AYAs, with an AAPC of 5.2%, which was more than double that of global males (2.4%) and significantly higher than that of Chinese females (2.1%), revealing pronounced sex disparities.

In terms of temporal patterns, global incidence rates for males briefly accelerated between 2003 and 2009 (APC = 4.04%), followed by a stabilization. In contrast, incidence rates for Chinese males demonstrated sustained and rapid growth: 3.27% during 1990–2000, surging to 8.40% during 2000–2010, nearly three times that of males globally during the same period. This high rate persisted between 2010 and 2018 (APC = 5.25%), with a slight decline between 2018 and 2021 (APC = 1.36%).

The global sex gap in the AAPC remained relatively modest (Δ = 0.6%), while the gap widened significantly (Δ = 3.1%) in China, suggesting that Chinese males may be exposed to stronger or more specific risk factors, necessitating targeted prevention and intervention efforts.

#### Chinese female incidence showed pronounced cyclical fluctuations with a “stagnation–rebound” pattern.

The global female incidence remained relatively stable, especially after 2010, and the AAPC was consistently lower than that of males (0.86% vs. 2.13%). In contrast, Chinese females exhibited marked fluctuations characterized by cycles of stagnation followed by rebounds. This “stagnation–rebound” trend underscored the importance of establishing stable, long-term, and adaptive prevention and management strategies tailored to females.

### APC analysis results for TC incidence among AYAs

#### APC analysis of TC incidence among AYAs in China demonstrated a strong age‐dependent increase in risk.

**Fig 5:**
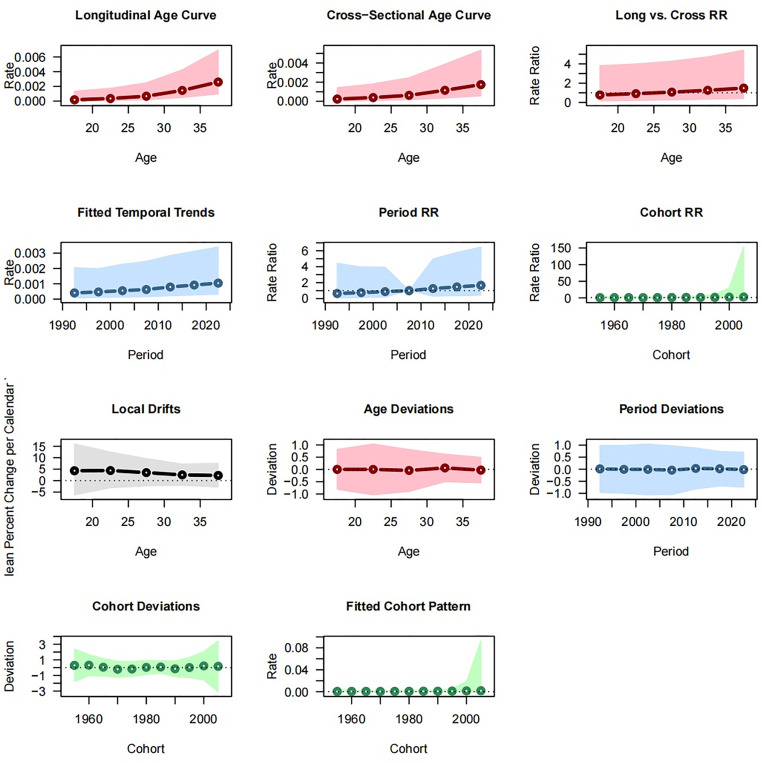
Age-period-cohort analysis of thyroid cancer incidence in the Chinese AYA population.

**Fig 6:**
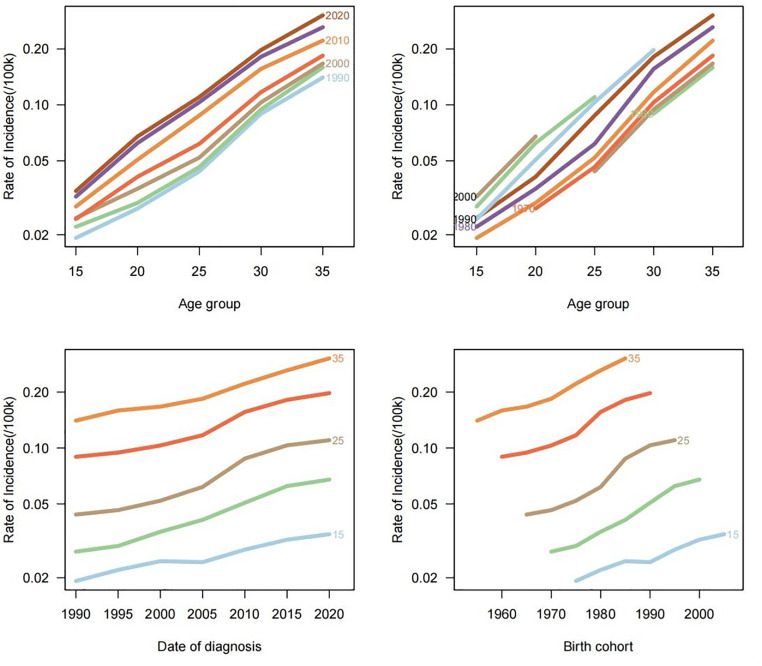
APC analysis of thyroid cancer incidence among Chinese AYAs based on five-year age groups, periods, and birth cohorts.

Fig 5 depicts the APC analysis of TC incidence among Chinese AYAs, while Fig 6 details corresponding stratified effects with 5-year age intervals. Both the longitudinal and cross-sectional age curves rose steeply with advancing age; individuals aged 35–39 years experienced an incidence rate of 0.003 per 100,000 (95% CI: 0.001–0.007), which was three times higher than that observed in the 15–19 year old group. The longitudinal age trend (LAT) parameter of 0.134 (95% CI: 0.048–0.220; *p* < 0.05) indicated that each additional year of age was associated with a 14.3% increased risk of TC. Local drift estimates further reinforced this pattern, showing annual percentage changes of 2.3%–2.5% for the 30–39 year old cohort. Goodness-of-fit tests (age deviation: −0.027, 95% CI: −0.545–0.491) confirmed that the model accurately captured the elevated incidence of older AYAs. Together, the consistent upward trends in age curves, statistically significant age parameter, and increasing relative risks (RR) supported a marked age-driven increase in TC incidence among Chinese AYAs.

#### Period effects were modest and statistically nonsignificant.

From 1990 to 2020, the fitted period trend remained essentially flat (approximately 0.001 per 100,000), although an upward shift was suggested after 2010; for example, the period RR in 2017 reached 1.467 (95% CI: 0.372–5.782). However, the wide CI, a net drift of 3.314% per year (*p* = 0.1513), and a nonsignificant Wald test (*p* = 0.8641) indicated that calendar‐period factors exerted, at most, a weak influence on incidence trends. A closer examination of the 2010–2015 period deviation (0.033; 95% CI: −0.817–0.882) revealed that the modest increase may reflect the expanded use of high-resolution ultrasound rather than genuine epidemiological shifts, with overdiagnosis potentially confounding any true period effects.

#### Cohort effects show a nonsignificant but suggestive pattern of increased risk among more recent birth cohorts.

The cohort RR for those born in 2000 was 2.317 (95% CI: 0.177–30.37) compared to the 1980 cohort, although this difference was not statistically significant (Wald *p* = 0.9834). The cohort age trend (CAT) parameter of 0.101 (95% CI: 0.033–0.170; *p* < 0.05) implied a 10.1% higher log‐risk of TC in later cohorts, suggesting that early‐life environmental exposures may have lasting biological effects. Nonetheless, the cohort deviation of 22.2% (95% CI: −1.585–2.029) for the 2000 cohort relative to the model predictions lacked precision, underscoring the need for additional data to confirm any true birth cohort influence.

### BAPC projections

To assess the predictive validity of the BAPC model, forecasts for 2016–2021 were compared with observed TC data among Chinese AYAs. In 2021, predicted ASMR (0.04, 95% CI: 0.03–0.05), ASDR (3.34, 95% CI: 2.97–3.71), ASPR (13.68, 95% CI: 11.95–15.41), and ASIR (1.43, 95% CI: 1.25–1.61) closely matched observed rates (0.04, 3.39, 13.11, and 1.44, respectively), all within the corresponding 95% CIs, indicating that the BAPC model provides accurate short-term predictions.

**Fig 7:**
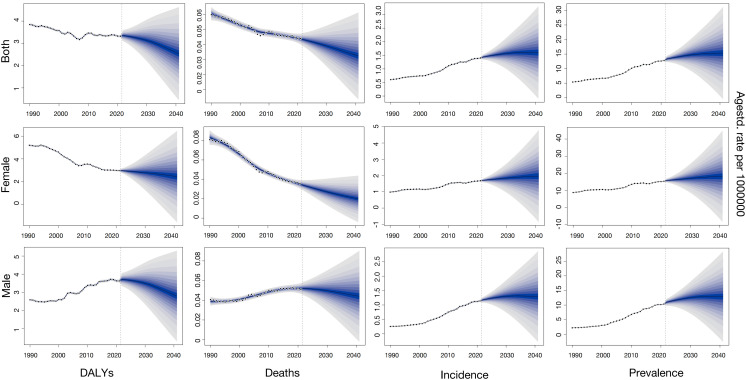
Diverging trends in thyroid cancer burden among Chinese AYAs: 20-year BAPC projections (2022–2041). **(A)** Projected age-standardized incidence rates show a continued rise overall, with females bearing a consistently and significantly higher burden than males. **(B)** Projected mortality rates remain very low, showing a slight decline for females and stability for males.

Building on this validation, the BAPC modeling projected the ASR of TC among Chinese AYAs from 2022 to 2041, with detailed trajectory forecasts presented in Fig 7. The projections revealed a pronounced “two-up, one-down” pattern: age-standardized incidence and prevalence rates are expected to continue increasing, age-standardized DALYs will gradually decline, and mortality will remain stable. Notably, significant sex differences persist, and the widening uncertainty in long-term forecasts, particularly beyond 2040, calls for cautious interpretation.

#### Overall population trends.

The TC burden of AYAs in China from 2022 to 2041 was projected using the BAPC model. Age-standardized DALYs are expected to decline from 3.35 per 100,000 in 2022 to 2.54 (95% CI: 0.46–4.62) per 100,000 by 2041, indicating a gradual reduction in healthy life-years lost. Mortality will remain stable at approximately 0.03 (95% CI: 0.01–0.05) per 100,000 over the period. In contrast, the incidence is forecasted to increase from 1.44 to 1.60 (95% CI: −0.09–3.29) per 100,000 and the prevalence from 13.33 to 15.33 (95% CI: −0.41–31.07), per 100,000, reflecting the ongoing pressures from both new diagnoses and an expanding survivor population.

#### Sex-specific trends.

Female AYAs carry a higher absolute burden: their standardized DALYs will decrease from 2.97 to 2.45 (95% CI: −1.55–6.45) per 100,000, while incidence will increase from 1.70 to 1.97 (95% CI: −0.85–4.79) per 100,000 and prevalence from 15.81 to 18.30 (95% CI: −8.12–44.72) per 100,000. Male AYAs will experience a lighter but accelerating burden: DALYs will drop from 3.71 to 2.79 (95% CI: 0.30–5.28) per 100,000, yet incidence will increase from 1.18 to 1.29 (95% CI: −0.28–2.86) per 100,000 and prevalence from 10.99 to 12.89 (95% CI: −2.50–28.28) per 100,000. Mortality projections show a slight decline for females—from 0.03 to 0.02 (95% CI: 0.0004–0.0396) per 100,000—and stability for males—from 0.04 to 0.04 (95% CI: 0.0008–0.0792) per 100,000.

## Discussion

### Global trends and the “Incidence-Mortality Divergence” in Chinese AYAs

This study systematically elucidated the global heterogeneity of the TC burden among Chinese AYAs, along with the potential underlying drivers. Overall, TC in Chinese AYAs demonstrated a distinctive epidemiological pattern of “accelerated incidence with improved mortality.” From 1990 to 2021, the ASIR increased by 152.6%, significantly exceeding the global growth rate, while both mortality and DALYs declined. This revealed a triad of high incidence, low mortality, and mild disability, indicating epidemiological heterogeneity. The increasing trend in TC incidence may be driven by the widespread use of imaging-based screening and improvements in diagnostic sensitivity, which have raised concerns about overdiagnosis [25–28]. Notably, the GBD study includes all histopathologically confirmed TC cases in its estimations, encompassing asymptomatic subclinical cases identified through screening. This may lead to an overestimation of incidence rates compared to the true biological increase in disease occurrence.

### Drivers of overdiagnosis and the role of clinical advances

From a disease burden perspective, despite the sharp rise in incidence, DALYs have not followed the same upward trajectory and have, in fact, continued to decline. This divergence may reflect a dual underlying mechanism. On the one hand, overdiagnosis has led to the inclusion of a substantial number of low-risk cases—particularly subclinical cancers—which rarely result in death or functional impairment and therefore contribute minimally to DALYs. On the other hand, improvements in early diagnosis and treatment—such as standardized surgical approaches and postoperative care—have effectively improved patient outcomes. While overdiagnosis significantly influences incidence estimates, the sustained decline in TC mortality among Chinese AYAs objectively reflects the positive impact of advances in clinical management and care systems.

### Sex disparities in incidence: The female predominance and its implications

Sex-stratified analyses revealed significant heterogeneity. Globally, females have consistently higher incidence rates of TC than males (2021 ASIR: 2.38 vs. 0.88 per 100,000), with the female incidence approximately 2.7 times that of males.This gender disparity is likely influenced by a combination of biological and social factors. First, the interaction between the thyroid and female reproductive hormones is considered a key mechanism in the development of thyroid disorders and nodule formation [29]. After puberty, the incidence rates increase sharply in females and decline after menopause, implicating endocrine hormones in the pathogenesis of the disease. Notably, TC is the second most common malignancy during pregnancy after breast cancer, and nearly 10% of cases in females of reproductive age are diagnosed during pregnancy or early postpartum [30]. Accumulating evidence suggests that estrogen and its receptors promote TC cell proliferation and may affect disease progression via regulation of angiogenesis and metastasis [31]. Additionally, Hashimoto’s thyroiditis (HT) demonstrates a higher prevalence in females and is a well-established risk factor for TC [32]. On the other hand, sociobehavioral factors may further contribute to the rising incidence among females. Women typically undergo more frequent healthcare visits than men—for example, for reproductive health assessments or perimenopausal management—which increases the likelihood of thyroid screening and, consequently, the risk of overdiagnosis [33]. In recent years, the overdiagnosis of TC has grown rapidly worldwide and has emerged as a significant public health concern. In China, this issue is particularly pronounced among women: studies have shown that between 2008 and 2012, approximately 260,000 female TC cases were considered overdiagnosed, accounting for 87% of all female diagnoses during that period [34]. Although these cases generally do not lead to clinical symptoms or affect survival, they can result in unnecessary medical interventions, psychological distress, and substantial economic burden. Therefore, it is imperative to refine current TC screening strategies—particularly those targeting women—to reduce overdiagnosis, avoid overtreatment, and ensure more efficient allocation of healthcare resources.

### Underlying drivers in young males: Environmental and metabolic contributors

Although the absolute incidence among young males remains significantly lower than that among females worldwide, the rate of increase is accelerating, particularly in Chinese males. In China, the rate of increase in male incidence has accelerated markedly, with the EAPC nearly three times higher than that in females. The rise in TC prevalence among young Chinese males was predominantly driven by epidemiological factors, accounting for 88.6% of the observed increase, which was substantially higher than the 61.2% observed globally in males. Increasing attention has been paid to environmental pollution and chemical exposures, which may also serve as potential contributing drivers [35,36]. Long-term exposure to polybrominated diphenyl ethers (PBDEs) is a suspected trigger for endocrine-related malignancies [37], and BDE-209 is the most prevalent PBDE detected in environmental samples [38]. Long-term exposure to PBDEs has been established as an inducer of endocrine-related malignancies [39]. Notably, BDE-209—the predominant PBDE congener in environmental media [36]—reached concentrations of 3,100 ng/g lipid weight in serum samples from Chinese e-waste dismantlers, representing the highest occupational exposure level documented at that time [39]. Crucially, a study [40] demonstrated significantly elevated serum BDE-209 levels in male workers compared to females (*p* < 0.001), with analogous trends observed for BDE-100 (*p* = 0.029) and BDE-183 (*p* < 0.001). This finding aligns with prior human biomarker studies confirming systematically higher PBDE body burdens in males across blood [41,42] and adipose tissue matrices [43]. However, this observed disparity may primarily reflect differential occupational exposure patterns. Moreover, a stuty has found that occupational exposure to ionizing radiation significantly elevates the TC risk (OR: 1.61, 95% CI: 1.27–2.04, *p* < 0.001), with a greater effect observed in males than in females (male vs. female OR: 1.74 vs. 1.30) [44]. However, current evidence on the causal relationship between environmental exposure and TC remains limited, underscoring the need for longitudinal and mechanistic research [35].

Beyond external exposures, metabolic risk factors are progressively accumulating in the young male population.High body mass index (BMI), a well-established risk factor for TC [22,45–47], has become an increasingly significant burden among younger populations [48]. Between 1990 and 2021, the global deaths and DALYs attributable to a high BMI among AYAs has increased by 109% and 141%, respectively, with faster growth observed in males than in females [49]. In the Chinese population, both the prevalence of overweight and obesity are higher in men than in women, with male obesity peaking at ages 35–39 [50,51]. This trend may further amplify the “catch-up effect” in incidence among young males, forming the epidemiological foundation for the rising burden in this group.

### Prognostic vulnerability of male patients

In comparison to the rapidly increasing incidence, young males are likewise confronted with considerable challenges regarding health outcomes. In terms of health outcomes, while the global burden of TC in young males has increased modestly, China has displayed a distinct pattern. The mortality EAPC for young males was + 1.26%, whereas females experienced a decline (EAPC = −2.73%), highlighting the disadvantaged status of males regarding disease outcomes. Sex has been consistently identified as an independent prognostic factor in TC [52], and males have poorer overall survival (OS), disease-specific survival (DSS), and disease-free survival (DFS) [53–56]; are more likely to be diagnosed at an advanced stage; and more frequently undergo lymph node dissection and radioactive iodine (RAI) therapy [55,57] than females. They also face a higher risk of recurrence [58–60]. Known risk factors such as radiation exposure and iodine intake are insufficient to fully explain this pronounced sex disparity in prognosis [3,18], suggesting that sex-specific biological mechanisms, clinical pathways, and social behaviors may contribute to outcomes and require further exploration.

Current evidence suggests that the poorer prognosis of TC in males results from the combined effects of delayed diagnosis, more aggressive pathological features, and pro-metastatic molecular mechanisms. At the time of diagnosis, male patients are more frequently in stage III or IV, with the proportion of advanced-stage disease being approximately 40% higher than in females [61,62]. The primary tumors in males are larger and more invasive, with a significantly greater mean tumor diameter (>2 cm vs. < 1.5 cm) and a 1.8-fold higher rate of extrathyroidal extension [63,64]. The central lymph node metastasis rate reaches 53.6% in males, compared to 40.0% in females, and males are also more likely to develop distant metastases, particularly to the lungs and liver [61,65,66]. Histological subtypes in males tend to be more malignant, with higher proportions of follicular carcinoma and Hürthle cell carcinoma—approximately 30% more than in females—which are prone to hematogenous spread [61]. Aggressive variants, such as tall-cell papillary thyroid carcinoma, are also significantly more common in males [67,68]. These findings indicate that males exhibit more aggressive clinical and biological characteristics of TC, warranting greater vigilance in clinical practice.

In conclusion, TC among Chinese AYAs has undergone a unique epidemiological transition over the past three decades, with a particularly marked “catch-up burden” observed in males. Although the overall mortality rates have declined, the challenges posed by the high incidence and rising prevalence underscore the urgent need for precise public health strategies. These should encompass optimized screening protocols, environmental exposure mitigation, obesity interventions, and sex-specific disease management to effectively address the escalating disease burden and improve outcomes in vulnerable populations.

## Limitations

This study leveraged the GBD 2021 dataset to analyze AYA TC trends, sex differences, and drivers, globally and in China, from 1990 to 2021, and employed multiple models to forecast the future burden. Nevertheless, this study had some limitations. First, although the GBD provides standardized methodology and broad coverage, its reliance on secondary data and modeling may introduce bias, especially when primary data are incomplete or of variable qualitydue to diagnostic variability across regions and changes in screening practices. Moreover, the GBD framework cannot distinguish between true increases in incidence and overdiagnosis, potentially leading to overestimated disease burden in some populations. Second, the APC and BAPC models, although powerful for trend identification, cannot fully adjust for unmeasured confounders or isolate the impact of specific exogenous risks (e.g., environmental chemicals and lifestyle factors). Third, we did not stratify patients by TC histological subtypes (e.g., papillary vs. follicular) or explicitly model changes in clinical practice (e.g., RAI use and screening guidelines), which could deepen the clinical interpretability. Future research should integrate registry‐level, individual‐patient data and detailed exposure metrics to further refine our understanding of AYA TC epidemiological heterogeneity and inform precise prevention strategies.

## Conclusions

Over the past three decades, the burden of TC among Chinese AYAs has changed markedly, with an accelerated incidence and prevalence, along with improved mortality and DALYs. From 1990 to 2021, the ASIR increased by 152.6% in China, far outpacing global levels, while mortality and DALYs declined, yielding a distinctive “high incidence–low mortality–low disability” profile. Decomposition analysis indicated that this surge in incidence was primarily driven by epidemiological shifts rather than demographic changes. Notably, the annual incidence growth rate among male AYAs in China was three times that of females, and male DALYs increased as female DALYs declined, demonstrating pronounced sex heterogeneity. Joinpoint and APC modeling showed that the upward trend in China began earlier and was steeper and more volatile than global patterns, with the age effect indicating a higher risk among older AYAs. BAPC projections suggest that by 2041, the incidence and prevalence among Chinese AYAs will continue to rise, mortality will remain stable, and lost healthy life years will decrease. Although females will still bear a higher absolute burden, the accelerated incidence among males warrants particular attention. These findings underscore the need for more precise, forward-looking public health strategies, such as optimized screening, targeted environmental and lifestyle interventions, and sex-specific management, to effectively curb the ongoing rise in the TC burden among the youth in China.
